# Second-Line Irinotecan, Leucovorin, and 5-Fluorouracil for Gastric Cancer Patients after Failed Docetaxel and S-1

**DOI:** 10.1155/2016/6857625

**Published:** 2015-12-29

**Authors:** Joo Young Jung, Min-Hee Ryu, Baek-Yeol Ryoo, Boram Han, Ji Woong Cho, Man Sup Lim, Hyun Lim, Ho Suk Kang, Min-Jeong Kim, Hong Il Ha, Hunho Song, Jung Han Kim, Hyeong Su Kim, Yoon-Koo Kang, Dae Young Zang

**Affiliations:** ^1^Department of Internal Medicine, Hallym University Medical Center, Hallym University College of Medicine, Anyang-si, Gyeonggi-do 14068, Republic of Korea; ^2^Department of Oncology, Asan Medical Center, University of Ulsan College of Medicine, Seoul 05505, Republic of Korea; ^3^Department of Surgery, Hallym University Medical Center, Hallym University College of Medicine, Anyang-si, Gyeonggi-do 14068, Republic of Korea; ^4^Department of Radiology, Hallym University Medical Center, Hallym University College of Medicine, Anyang-si, Gyeonggi-do 14068, Republic of Korea

## Abstract

*Background.* This retrospective study aimed to assess the efficacy and toxicities of second-line chemotherapy with irinotecan, leucovorin, and 5-fluorouracil (5-FU) in metastatic gastric cancer (MGC) patients previously treated with docetaxel and S-1 with or without oxaliplatin (DS/DOS).* Patients and Methods.* We reviewed the data of patients who had previously been treated with first-line DS/DOS and received biweekly irinotecan-based chemotherapy (FOLFIRI/IFL) between October 2004 and November 2011.* Results.* A total of 209 cycles were administered to 35 patients, with a median of 4 (range, 1–22) cycles each. The overall response rate in 29 response-assessable patients was 17.2%, including 2 complete and 3 partial responses. The median progression-free and overall survivals were 3.81 (95% confidence interval [CI], 1.82–5.80) months and 6.24 (95% CI, 1.44–11.04) months, respectively. The major grade 3/4 toxicity was neutropenia (8.6%).* Conclusion.* FOLFIRI/IFL chemotherapy showed modest antitumour activity and tolerable toxicities in DS/DOS-treated MGC patients.

## 1. Introduction

Gastric cancer is a common malignancy and a major cause of cancer-related death worldwide [1]. Recent advances in early diagnosis, surgery, and chemotherapy have helped cure patients with gastric cancer. However, many patients either experience a relapse after curative resection or are diagnosed with locally advanced or metastatic disease [2].

Palliative systemic chemotherapy prolongs overall survival (OS) and improves quality of life (QOL) for relapsed or metastatic gastric cancer (MGC) patients compared to best supportive care [3]. However, drugs selected for first-line therapy can vary in efficacy depending on regional or racial differences [3–10]. Recently, modification to the oral fluoropyrimidine regimen for advanced gastric cancer (AGC) has been attempted to improve efficacy [11–15], and we previously demonstrated the efficacy and tolerability of first-line treatment with docetaxel and S-1 with oxaliplatin (DOS) or without oxaliplatin (DS) in AGC [13–15].

The efficacy of first-line chemotherapy in AGC is usually modest. Furthermore, the median OS after failure of first-line chemotherapy is ≤4 months without additional anticancer treatment [15–18]. Second-line palliative chemotherapy has shown to have OS benefits in 3 phase III trials [16–18] and in a recent meta-analysis [15]. These studies demonstrated that monotherapy with either irinotecan or docetaxel was an effective and acceptable regimen for MGC in a second-line setting.

Many studies investigated irinotecan-based chemotherapy as monotherapy or combination regimens, including as salvage chemotherapy [19–21]. They showed favourable efficacy with tolerable safety profiles, which is essential for second-line chemotherapy for MGC. Hence, we postulated that FOLFIRI/IFL as salvage therapy may benefit MGC patients who had been previously treated with first-line DS/DOS. To our knowledge, there have been no previous studies with second-line irinotecan, 5-fluorouracil (5-FU), and leucovorin combination regimens for MGC patients previously treated with DS/DOS.

Herein, we investigated the efficacy and toxicity of second-line irinotecan-based combination chemotherapy in first-line DS/DOS-treated MGC patients.

## 2. Material and Methods

### 2.1. Study Population

Patients with MGC who previously failed first-line DS/DOS and had received irinotecan-based combination chemotherapy as second-line treatment at two institutions (Hallym University Medical Center and Asan Medical Center, Korea) between October 2004 and November 2011 were included in this retrospective study. All data were collected from the electronic medical records. This study protocol was approved by the Institutional Review Boards of both participating institutions and was conducted in accordance with the principles of the Declaration of Helsinki.

### 2.2. Treatment Schedule

Two biweekly irinotecan-based regimens were used. FOLFIRI consisted of irinotecan (150 mg/m^2^ in a 2 h infusion) on day 1, then leucovorin (200 mg/m^2^ in a 2 h infusion) and 5-FU (400 mg/m^2^ bolus followed by 600 mg/m^2^ in a 22 h continuous infusion) on days 1 and 2 every 2 weeks. IFL consisted of irinotecan (150 mg/m^2^ in a 2 h infusion) on day 1, leucovorin (20 mg/m^2^ intravenous bolus infusion) on days 1 and 2, and 5-FU (500 mg/m^2^ as an intravenous bolus infusion) on days 1 and 2, every 2 weeks. IFL was administered to the patients who were previously treated with DS (October 2004 to July 2007), and FOLFIRI was administered to those previously treated with DOS (July 2007 to January 2010). IFL was replaced by FOLFIRI based on previously published results [19, 22]. Chemotherapy was administered until disease progression or unacceptable toxicity.

### 2.3. Efficacy and Safety Assessments

Baseline evaluation included medical history, physical examination, complete blood counts (CBC), blood chemical analysis, and radiological examinations; all but radiology were also performed prior to each cycle. Tumour assessments using chest and abdominopelvic computed tomography (CT) were performed every four cycles or when disease progression was suspected. The response to chemotherapy was assessed according to the Response Evaluation Criteria in Solid Tumours version 1.0. Toxicities were assessed according to the National Cancer Institute Common Toxicity Criteria version 3.0.

### 2.4. Statistical Analysis

All patients who received at least one cycle of an irinotecan-based regimen were included in safety analyses. Efficacy was analysed in patients who received at least two cycles of chemotherapy. PFS was measured from the initiation of FOLFIRI/IFL chemotherapy until the time of first occurrence of progression, death from any cause, or the date of last follow-up. OS was based on the interval between the first day of treatment and the time of death for any reason or the date of the last follow-up visit. PFS and OS were determined using the Kaplan-Meier method. The differences between the survival curves were analysed using the log-rank test.

## 3. Results

### 3.1. Patient Characteristics

This study included 35 patients from two institutions treated between October 2004 and November 2011. Six patients were not included in the response evaluation but were evaluated for safety analysis because they each received only one cycle of treatment; the remaining 29 were evaluated for efficacy. Baseline patient characteristics are presented in Table 1.

### 3.2. Treatment

IFL was administered to DS-treated patients (25.7%) and FOLFIRI was used for those treated with DOS (74.3%). A total of 209 cycles of chemotherapy were conducted, with a median of 4 cycles per patient (range, 1–22 cycles). However, patients with early progression (*n* = 3) or clinical impairment or toxicity (*n* = 3) received only one cycle. The mean and median relative dose intensity for irinotecan during the total treatment cycles were 65.3 mg/m^2^/week (87.1% of the planned dose) and 75.0 mg/m^2^/week (100% of the planned dose), respectively. There was no initial dose reduction. Of the 26 patients who received ≥3 cycles of chemotherapy, 9 (34.6%) underwent at least one level of dose reduction, and another 9 (34.6%) experienced treatment delay of >1 week because of adverse events, including neutropenia (*n* = 7), oral mucositis/anorexia/gastrointestinal infection (*n* = 1), and acute appendicitis (*n* = 1).

### 3.3. Efficacy

**Figure 1:**
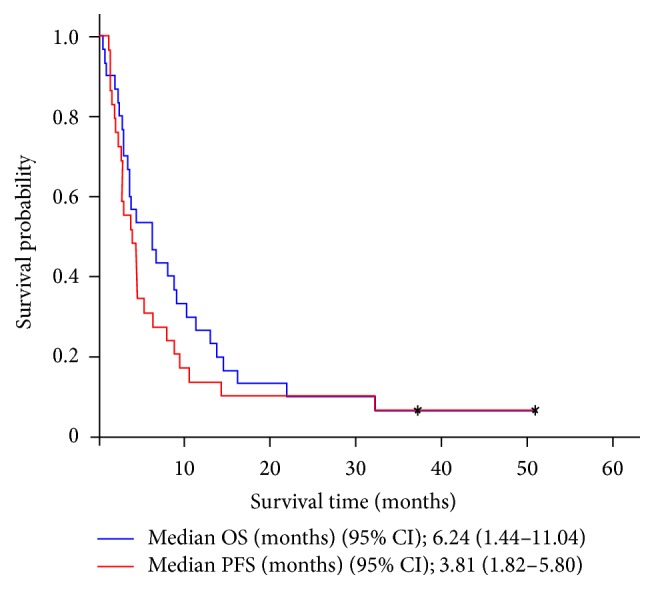
Kaplan-Meier plot of progression-free survival (PFS) and overall survival (OS).

**Figure 2:**
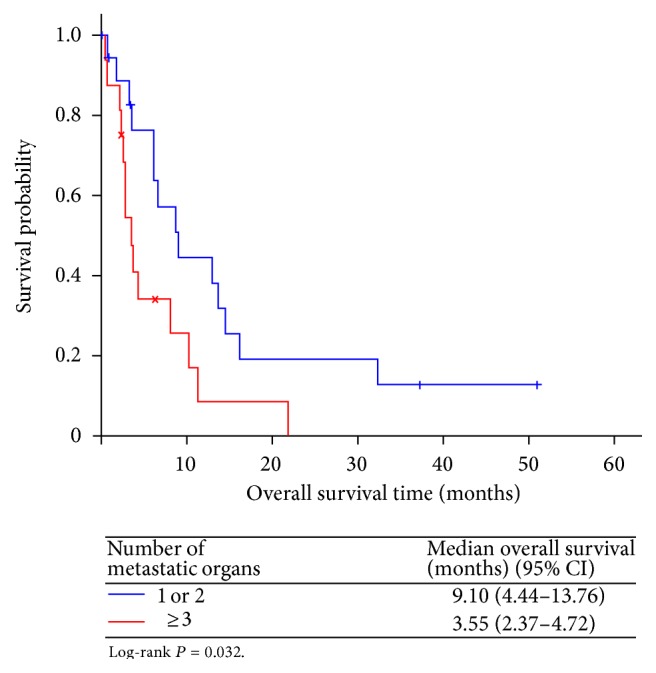
Overall survival (OS) stratified by the number of metastatic organs.

**Figure 3:**
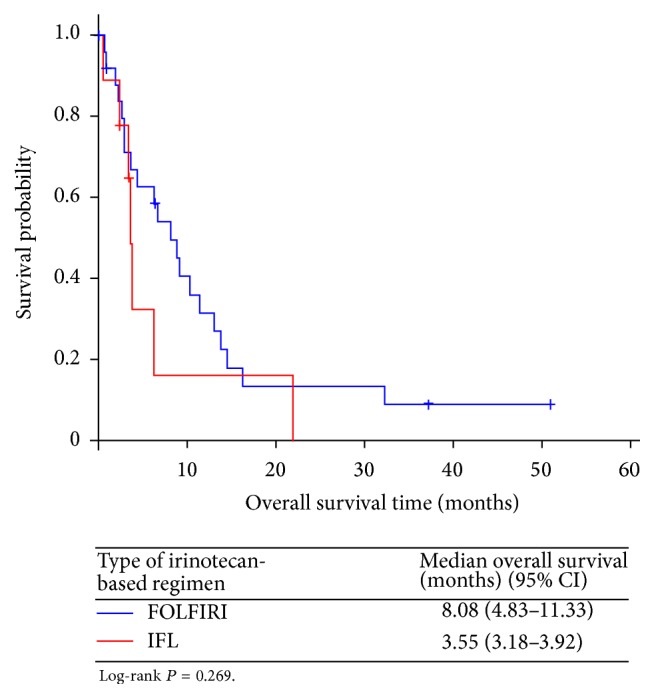
Overall survival (OS) stratified by the type of irinotecan-based regimen.

Of the 29 evaluable patients, 2 (6.9%) achieved complete response, 3 (10.3%) had partial response, 12 (41.4%) demonstrated stable disease, and 12 (41.4%) had progressive disease (Table 2). In the 2 patients with complete response, one had hepatic and nodal metastasis and the other had peritoneal metastasis. The overall response rate (ORR) was 17.2% and the disease control rate (DCR) was 58.6%. The median PFS was 3.81 (95% confidence interval [CI], 1.82–5.80) months for “per protocol” patients, and the median OS was 6.24 (95% CI, 1.44–11.04) months, with a 1-year survival rate of 24.8% (Figure 1). Moreover, the number of organs with metastases (<3 versus ≥3) affected OS (hazard ratio [HR] 0.436 [95% CI, 0.200–0.948], *P* = 0.036) (Figure 2). There were no significant differences in OS between patients treated with FOLFIRI (8.08 [95% CI, 4.83–11.33] months) and those treated with IFL (3.55 [95% CI, 3.18–3.92] months) (HR 0.612 [95% CI, 0.253–1.476], *P* = 0.269) (Figure 3).

Fourteen patients received additional treatment after completion of irinotecan-based chemotherapy; 10 patients received FOLFOX (oxaliplatin, 5-FU, and leucovorin) and 3 received chemotherapy using taxanes with or without cisplatin (1 patient each received paclitaxel plus cisplatin, docetaxel plus cisplatin, and cabazitaxel).

### 3.4. Treatment-Related Adverse Events

All 35 patients were evaluable for toxicity (Table 3). In terms of haematological toxicities, the frequencies of grade 3 neutropenia, anaemia, and febrile neutropenia were 9%, 3%, and 3%, respectively. Most of the observed nonhaematological toxicities were grades 1-2, with the exception of grade 3 anorexia observed in 1 patient. There were no grade 4 toxicities.

## 4. Discussion

Some studies have evaluated DS-based first-line chemotherapy and irinotecan-based second-line chemotherapy for MGC [13–17, 19–21, 23]. However, few have specifically focused on FOLFIRI/IFL chemotherapy in gastric cancer patients previously treated with docetaxel or S-1, and only as monotherapy after administration of docetaxel [24], S-1 [25], or DCF [26]. Our study included 35 patients who had been previously exposed to DS/DOS. The median PFS and OS after treatment with FOLFIRI/IFL were 3.81 and 6.24 months, respectively (Figure 1), and the treatment-related toxicities were tolerable (Table 3). This is the first study of FOLFIRI/IFL as second-line palliative chemotherapy in DS/DOS-treated MGC patients.

According to numerous phase II and 3 phase III studies on the efficacy of irinotecan and docetaxel in second-line settings [16–18, 21], both agents are effective and safe for salvage chemotherapy in MGC. We chose irinotecan over docetaxel because the former has been shown to be effective, and all of the patients included in our study had previously been exposed to the latter.

In studies of the efficacy of irinotecan-based chemotherapy in S-1 [24] or docetaxel [26] treated MGC, the ORR, PFS, and OS were 15.4–42.0%, 2.9–6.3 months, and 7.0–8.9 months, respectively [20, 24–26]. In our study, the ORR and DCR of FOLFIRI/IFL were 15.6% and 53.1%, respectively (Table 2), and the median PFS and OS were 3.81 (95% CI, 1.82–5.80.42) and 6.24 (95% CI, 1.44–11.04) months, respectively (Figure 1). Our results are therefore comparable to those of previous studies of second-line irinotecan-based regimens [21, 27].

Effective salvage therapy must balance symptom control and toxicity to preserve QOL. The frequency of grade 3/4 haematological toxicity (especially neutropenia) with irinotecan-based regimens was 30–50% in previous studies [20, 21, 24], usually resulting in dose modification and chemotherapy delay. In our study, the incidence of grade 3 haematological toxicity with FOLFIRI/IFL was lower than that of other studies investigating second-line treatment for MGC. There were no grade 4 toxicities in our patients. While the mean dose intensity of irinotecan in all cycles was ≤90% (87.0%), 9 (31.0%) of 29 response-evaluable patients were treated with ≥8 cycles; the efficacy was not inferior to those of previous similar studies. Furthermore, 2 patients achieved complete response and survived until the last follow-up, and 1 underwent 22 cycles of treatment without progression or serious adverse events. These data suggest that second-line FOLFIRI/IFL for MGC can accomplish a durable response with acceptable toxicity through optimal dose and schedule modification.

As mentioned above, we changed the irinotecan-based regimen from IFL to FOLFIRI in patients previously treated with DS. Although the doses of the 3 drugs in the IFL regimen were lower than those in the FOLFIRI regimen and all IFL drugs were infused within 2 hours, we observed no significant differences in efficacy between the different regimens (Figure 3). This may be owing to our small cohort size, although it is also possible that there was no significant heterogeneity in efficacy among the various irinotecan-based regimens. These findings suggest that both salvage regimens are options in MGC; prospective studies with more participants are required to confirm the differences between them.

In recent years, several studies reported the efficacy of vascular endothelial growth factor receptor- (VEGFR-) targeted agents in AGC patients. VEGFR-2 is particularly overexpressed in gastric cancer tissue, especially in presence of lymph nodal metastases [28].

Recent trials demonstrated that anti-VEGFR-2 monoclonal antibody, ramucirumab, can increase the PFS and OS in second-line setting as monotherapy [29] and combination with chemotherapy [30]. The recent placebo-controlled, randomized phase III trial which compared weekly paclitaxel and ramucirumab over weekly paclitaxel alone (RAINBOW study) proved that the addition of a targeted agent to standard chemotherapy, in the second-line setting, can achieve survival advantage in OS (7.4 versus 9.6 months, *P* = 0.017). In addition, the clinical trials about the addition of ramucirumab to second-line FOLFIRI in patients with metastatic colorectal carcinoma demonstrated the positive effect on OS (13.3 versus 11.7 months, *P* = 0.0219) with manageable toxicities [31]. The results of these studies can support the theoretical background for the combination of ramucirumab with irinotecan-based chemotherapy in AGC. Therefore, we should consider the clinical trials about the ramucirumab plus FOLFIRI in second-line setting for the patients with AGC, in near future.

In the present study, we attempted to compare our patients' clinicopathological parameters to those of previous studies to determine prognostic factors of second-line chemotherapy for gastric cancer [32–34]. We found that patients with <3 metastatic sites at the time of enrolment had statistically longer OS than those with ≥3 metastatic sites (HR 0.436, 95% CI, 0.200–0.948, *P* = 0.036) (Figure 2). This was consistent with previous MGC studies.

This study had potential limitations because of its retrospective nature, the heterogeneity of the chemotherapy schedules, and the small number of patients. However, few previous reports address the benefits of irinotecan with fluoropyrimidine as salvage therapy after the failure of S-1 and taxane-based first-line chemotherapy in MGC patients. Furthermore, the heterogeneity of the chemotherapy schedules did not cause differences in the response or survival data in this study, as reported previously [21, 27].

In conclusion, a combined irinotecan, 5-FU, and leucovorin-based regimen is effective and acceptable salvage chemotherapy for MGC patients previously treated with DS/DOS.

## Figures and Tables

**Table 1:** Patient characteristics.

Characteristics	Number of patients	Percent (%)
Age (years), median (range)	50 (26–70)	
Gender		
Male/female	25/10	71.4/28.6
ECOG performance status		
0/1/2	10/23/2	28.6/65.7/5.7
Primary tumour location		
Upper 1/3 (cardia/fundus)	3	8.3
Mid 1/3 (body)	17	47.2
Lower 1/3 (antrum/pylorus)	9	25.0
Diffuse (entire stomach)	5	13.9
Unknown	1	5.6
Site of metastasis		
Nodal, distant	29	82.9
Peritoneum	19	54.3
Liver	9	25.7
Lung	4	11.4
Pancreas	3	8.6
Bone	3	8.6
Ureter	3	8.6
Ovary	2	5.7
Pleura	2	5.7
Number of metastatic sites		
<3/≥3	19/16	54.3/45.7
Histology		
Adenocarcinoma, well differentiated	4	11.4
Adenocarcinoma, moderately differentiated	7	20
Adenocarcinoma, poorly differentiated	17	48.6
Signet ring cell	5	14.3
Unclassified	2	5.7
Disease status		
Initially metastatic/recurrent	27/8	77.1/22.9
CEA level		
Normal/elevated/unknown	15/19/1	42.9/54.3/2.8
Haemoglobin level		
Normal/decreased	14/21	40.0/60.0
Surgery		
Gastrectomy/no gastrectomy	11/24	31.4/68.6
First-line regimen		
DS/DOS	9/26	25.7/74.3
Progressive disease after first-line chemotherapy		
During or ≤3 months after end of treatment	23	65.7
>3 months after end of treatment	12	34.3

DS: docetaxel plus S-1; DOS: docetaxel plus S-1 with oxaliplatin; ECOG: Eastern Cooperative Oncology Group; CEA: carcinoembryonic antigen.

**Table 2:** Response to treatment by per-protocol analysis of evaluable patients (*n* = 29).

Variable	Number of patients (%)
CR	2 (6.9)
PR	3 (10.3)
SD	12 (41.4)
PD	12 (41.4)
Overall response (CR + PR), %	17.2
Disease control (CR + PR + SD), %	58.6

CR: complete response; PR: partial response; SD: stable disease; PD: progressive disease.

**Table 3:** Treatment-related adverse events.

	Grade 1		Grade 2		Grade 3	
	Number	%	Number	%	Number	%
Haematological						
Neutropenia	2	5.7	8	22.9	3	8.6
Anaemia	6	17.1	6	17.1	1	2.9
Thrombocytopenia	4	11.4	0	0	0	0
Febrile neutropenia	0	0	3	8.6	1	2.9
Nonhaematological						
Anorexia	0	0	3	8.6	1	2.9
Emesis	2	5.7	3	8.6	0	0
Stomatitis	1	2.9	2	5.7	0	0
Diarrhoea	1	2.9	2	5.7	0	0
Neuropathy	2	5.7	0	0	0	0
